# Multi-Scale Organization of the *Drosophila melanogaster* Genome

**DOI:** 10.3390/genes12060817

**Published:** 2021-05-27

**Authors:** Samantha C. Peterson, Kaylah B. Samuelson, Stacey L. Hanlon

**Affiliations:** Department of Molecular and Cell Biology, University of Connecticut, Storrs, CT 06269, USA; samantha.c.peterson@uconn.edu (S.C.P.); kaylah.samuelson@uconn.edu (K.B.S.)

**Keywords:** *Drosophila*, topologically associating domain, insulator, pairing, transvection, B chromosome, cytogenetics

## Abstract

Interphase chromatin, despite its appearance, is a highly organized framework of loops and bends. Chromosomes are folded into topologically associating domains, or TADs, and each chromosome and its homolog occupy a distinct territory within the nucleus. In *Drosophila*, genome organization is exceptional because homologous chromosome pairing is in both germline and somatic tissues, which promote interhomolog interactions such as transvection that can affect gene expression in *trans*. In this review, we focus on what is known about genome organization in *Drosophila* and discuss it from TADs to territory. We start by examining intrachromosomal organization at the sub-chromosome level into TADs, followed by a comprehensive analysis of the known proteins that play a key role in TAD formation and boundary establishment. We then zoom out to examine interhomolog interactions such as pairing and transvection that are abundant in *Drosophila* but rare in other model systems. Finally, we discuss chromosome territories that form within the nucleus, resulting in a complete picture of the multi-scale organization of the *Drosophila* genome.

## 1. Introduction

Within the nucleus, the labyrinth of threads that comprise the genome does not, at first glance, appear to be organized. Indeed, when Walther Flemming initially described the dynamics of the material he called ‘chromatin,’ he designated one of the stages he observes as the ‘skein stage’ in an apparent reference to how the chromatin appears like a coiled and tangled skein of yarn [1]. From the observer’s perspective, one may conclude that order is eventually achieved as the cell moves into mitosis and the chromatin condenses into structured, discrete chromosomes; after their alignment and segregation into separate daughter cells, the chromosomes then dissolve back into a disorganized state within the nucleus.

From the chromosome’s point of view, however, the complete opposite is true. Despite its disordered appearance, chromatin is subject to complex, multi-scale organization that we have only started to understand. Surprisingly, as we touch on below, many of the integral associations that promote chromosomal organization disappear when chromatin condenses into individual chromosomes during cell division. Advancements in cytological techniques and DNA sequencing applications have challenged our definition of what it means for a genome to be organized and are revealing how chromosomes self-organize, as well as how and when they interact with their homolog and form discrete territories within the nucleus. Fueled by this acceleration of more sensitive tools for genomic investigation, the field of genome organization has exploded. 

**Figure 1:**
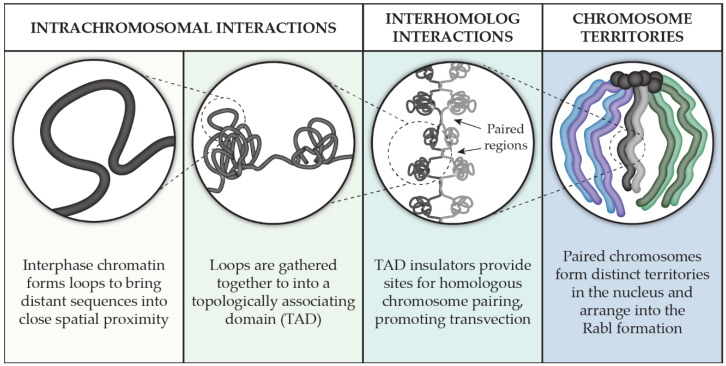
Genomic organization from the point of view of the chromosome.

Remarkable strides in understanding genome organization have taken place in many model systems from all three domains of life [2,3,4]. In this review, we chose to concentrate our focus on the multi-scale organization of chromosomes in the model system *Drosophila melanogaster* (Figure 1). We will pay particular attention to how chromosomes interact with themselves through our review of the key players involved in the formation and maintenance of topologically associating domains, or TADs. We then discuss our current knowledge of interhomolog interactions as it relates to pairing, transvection, and chromosome territories that are formed within the nucleus. In exploring chromosome associations on the nucleotide-to-nucleus scale, a larger picture of the genome’s organization from the perspective of the chromosome is revealed.

## 2. TADs in Drosophila

When not compacted into chromosomes during cell division, the genome resides in the nucleus as chromatin that is highly dynamic [5]. The flexibility of the DNA in this thread-like state enables chromosomes to bend and loop into complex conformations that establish tight spatial associations in three dimensions between genomic regions that can be quite distant at the sequence level. The result is self-interacting domains that are enriched in internal chromatin contacts, especially when compared to surrounding domains. The associations that form these domains are not random as their spatial relationship, or topology, remains mostly consistent after several cell divisions and across cell types, leading to the name topologically associating domains (TADs). Below, we discuss the methodology of TAD identification and how changes at the DNA level affect TAD dynamics. We will also go into detail about the proteins in *Drosophila* that are currently known to be important for TAD formation, regulation, and maintenance.

### 2.1. Sub-Chromosomal Organization and the Advent of TADs

The movement of interphase chromatin observed in live cells displays an interesting behavior: though it is free to move via diffusive random walk motion, this motion appears to be confined to smaller subregions within the nucleus [6]. Despite this restriction, chromatin is capable of long-range interactions that are recurrent and functional [7]. To understand the 3D organization of the genome and investigate the conformation of chromosomes in interphase, a new technique was developed: chromosome conformation capture, or ‘3C’ [8]. The underlying principle of this technique is to covalently link genomic sequences that are in close spatial proximity to one another and analyze the resulting fragments. This analysis can be on a small scale, such as probing for a single, specific interaction, as well as a genome-wide scale to examine the frequency of chromosomal contacts between all parts of the genome. The latter technique, generally referred to as ‘Hi-C,’ results in a visualization of chromatin organization on a genome-wide scale [9,10].

The arrival of advanced chromosome capture techniques that can detect chromosome contacts at the level of the whole genome revealed the complexity of chromosome interactions and transformed our view of genome organization. The chromosome contact map of domains in the *Drosophila* genome was soon after published [11], followed shortly by a pair of studies that examined chromosome contacts in mice and in human cells [12,13], the latter of which coined the term ‘TAD.’ Not only is this technique being applied routinely to compare genome organization across genotypes, cell types, and species but there has also been an emergence of variations of this technique that have the potential to add additional layers of information about the genome on top of the chromosome contact map [14,15]. For example, a new method called Hi-M has enabled the simultaneous capture of chromosome structure and transcriptional activity of a large (~350 kb) region of the genome in *Drosophila* embryos [16], and Micro-C has been applied in yeast to generate chromosome contact maps at nucleosome-scale resolution [17]. With the evolution of these techniques, our understanding of the functional aspects of genome organization will continue to grow.

### 2.2. Chromatin Context of TADs in D. melanogaster

*Drosophila melanogaster* has more than 4000 TADs, ranging in size from 3 to 460 kb [18]. The general structure of a *Drosophila* TAD consists of a transcriptionally active region close to the TAD border and an inactive region contained within the TAD [19]. Consistent with this model, the promoters and bodies of housekeeping genes and active marks are found at TAD boundaries, while H3K27me3, histone H1, and core histone H3 levels are depleted from TAD boundaries [20]. TAD boundaries also behave like classic insulator elements in that they bind known insulator proteins and are found between divergent promoters, as well as having a strong correspondence to interband regions on polytene chromosomes [21]. 

TADs have been thought to exhibit some form of hierarchy, with low-level, smaller TADs being contained within larger, high-level TADs [20]. This may be reflective of superimposed alternate folding patterns obtained by Hi-C data from populations of *Drosophila* cells, however, and modern single-cell Hi-C approaches have the potential to clarify this result [22,23]. Already, the single-cell Hi-C maps of *Drosophila* cell types have revealed more variability in interactions involving inactive chromatin, while interactions that were common in individual cells were composed of active chromatin. This makes sense as populations of the same cell type should have the same profile of active genes. Furthermore, boundaries that were conserved in one cell type tend to also be conserved in other cell types [22]. 

Despite the consistency of their boundary formation, TADs are likely dynamic in vivo as opposed to static [24,25]. In some situations, this flexibility can lead to unintended changes in TAD structure and regulation. For example, TADs associated with the nuclear lamina become more active and less compact and stretch toward the nuclear interior when the lamina is disrupted [26]. Additionally, chromatin is packed in the nucleus in interphase in inconsistent densities [27], and the chromatin architecture changes during the cell cycle [28], which has the potential to influence TAD behavior. In the *Drosophila* embryo, however, chromosome conformation and gene regulation were recently shown to be independent, and instead, TADs appear to fine-tune interactions between the promoters and enhancers of developmental genes [29,30]. 

Although there are many studies showing that chromatin marks are a prominent organizing factor in *Drosophila* [20,31,32], the exact mechanism of TAD formation remains unclear. It has been theorized that TADs can assemble themselves based on the level of histone acetylation: interactions are reduced between acetylated histones, while nonacetylated histones are more prone to interact, an effect that was sufficient for chromosome partitioning into TADs in computer models [31]. Separation of TADs may also be from methylation—or, more precisely, the lack thereof—as genomic regions with low H3K27me3 contain active housekeeping genes and are enriched for architectural proteins, whereas other regions with regulated genes, regardless of activity, have high or moderate levels of H3K27me3 [32]. It was predicted through polymer modeling that a dynamic loop extrusion mechanism is responsible for TAD formation, which consists of chromatin being pulled through a loop extruding factor (LEF) such as cohesin, until the LEF hits insulator proteins such as CTCF [24]. However, this process appears to be specific to mammals, with genome segregation being more essential to formation of TADs in *Drosophila* [19]. Interestingly, it has also been suggested that active transcription by RNA polymerase II plays a role in shaping *Drosophila* TADs, in conjunction with TAD boundary positions [33]. Hi-C at the sub-kb level suggests that epigenetic modifications are a factor for higher-order TAD folding, but the presence of pairs of insulator proteins was extremely predictive of a TAD boundary, and these may be more of a primary factor for the formation of individual TADs [18]. 

### 2.3. Insulator Proteins Involved in TAD Formation, Regulation, and Maintenance

In *D. melanogaster*, the overwhelming majority of TAD borders contain insulator elements [18,21].The proteins and the sequences to which they bind are integral for the chromosome architecture in *Drosophila*, and in the following section, we make an effort to collate our current knowledge of insulators in detail. *Drosophila* has many insulator proteins, which we have cataloged in this review (Table 1). One of these insulator proteins is a homolog of mammalian CTCF (dCTCF), which, at present, is the only known protein responsible for insulator activity in vertebrates. dCTCF has a similar structure to vertebrate CTCF, including 11 zinc fingers, and is just as widely expressed in all tissues and developmental stages in *Drosophila* as in vertebrates [34]. Unlike CTCF in mammals, which can act on its own, dCTCF needs other proteins to insulate regions of chromatin [35]. Recently, it was found that depleting dCTCF in embryos causes domain boundary defects near CTCF-binding sites, and dCTCF acts to insulate promotors and enhancers, rather than play a direct role in transcription regulation [36].

Other important insulator proteins in *Drosophila* include Boundary element-associated factor 32 (BEAF-32), Chromator, and CP190. In *Drosophila* embryos, Chromator, dCTCF, CP190, and BEAF-32 were found to be factors in partitioning physical domains in the genome [11]. BEAF-32 was first identified by immunostaining at the borders of polytene chromosomes [37]. BEAF-32 is preferentially found at borders of active regions, and dCTCF is found at borders of polycomb group-mediated regions [11]. BEAF-32, CP190, and Chromator are also found to be enriched at TAD boundaries [18]. Interestingly, Chromator is also enriched at dCTCF sites, suggesting it plays a role in chromatin organization [38]. CP190 intermediates interactions between insulator proteins and promoter sequences [39]. CP190 induces chromatin unfolding, possibly by recruitment of dCTCF, in both *Drosophila* and mammalian cells [40], and there are nearly 9000 CP190 binding regions in *Drosophila*, which overlap with two thirds of the dCTCF binding regions [41]. CP190 co-localizes with GAF, Su(Hw), cohesin, and E(Z) (a polycomb protein) [41]. Chromatin-linked adaptor for MSL proteins (CLAMP) appears to interact primarily with CP190, due to the disruption of its localization in the nucleus upon loss of CLAMP, and loss of CP190 correlates with much less recruitment of CLAMP to chromatin [42]. Chromator and chromatin remodelers are found to be increased at TAD boundaries [20]. Chromator, BEAF-32, RNA polymerase, JIL-1 kinase, and constitutively active (housekeeping) genes are enriched at the polytene chromosome interbands and active histone marks [19].

Suppressor of Hairy Wing (Su(Hw)) and Modifier of mdg4 (Mod(mdg4)) are also implicated in TADs. Mod(mdg4) interacts with Su(Hw), an insulator protein, and is required for enhancer–promoter blocking [43]. GAGA factor (GAF) is known to interact with Mod(mdg4) to regulate su(Hw) insulators [44]. GAF also interacts with chromatin remodelers, transcription machinery, and polycomb response elements (PREs) [45], and GAF is necessary for the maternal-to-zygotic transition in embryos [46]. GAF, like M1BP, has many functions throughout the genome. Another architecture protein, Motif 1 Binding Protein (M1BP), was first discovered to be a transcription factor that recruits Pol II to promoters [47]. M1BP binding motifs are found to be enriched at TAD boundaries [48]. Recently, M1BP was found to interact with insulator proteins, mainly CP190, but also Su(Hw) and Mod(mdg4), at TAD boundaries [49]. Depletion of M1BP correlates with genome compaction, which shows that M1BP plays a role in chromatin accessibility and genome organization [49]. 

Other well-known *Drosophila* architectural proteins include the product of the *zeste-white 5* gene (Zw5) [50], as well as Pita and zinc-finger protein interacting with CP190 (ZIPIC) [51]. Zw5 blocks enhancer–promoter interactions, but it is not strong enough to be an effective insulator by itself [50]. Pita and ZIPIC are insulators that directly interact with CP190 and help target it to promoters [51]. Pita was shown to interact with the BTB domain of CP190, whereas ZIPIC interacted with CP190 through a span of amino acids that included a part of its M domain [51]. 

Insulator proteins are often associated with specific epigenetics marks. H3K27me3-dense regions frequently have dCTCF, GAF, and Mod(mdg4) at their borders in the *Drosophila* genome [52]. dCTCF appears to associate with other insulator proteins at H3K27me3 region borders throughout the genome, including BEAF-32, Su(Hw), and CP190 [38,41]. dCTCF and CP190 are correlated with nucleosome loss and chromatin opening, and the loss of dCTCF or CP190 is correlated with an increase in repressive histone marks, such as H3K27me3 [41]. BEAF binding sequences are typically located very close to transcription start sites of active genes and are associated with RNA Pol II, H3.3, and H3K4me2, but they are not associated with Su(Hw) and dCTCF [53].

There are two classes of insulator complexes, each of which has distinct regulation mechanisms [52]. Class I insulators are characterized by the binding of BEAF-32, CP190, or dCTCF and act to protect regions of active chromatin from the encroachment of silent chromatin [52]. BEAF-32 has since become known as an insulator of high importance in *Drosophila* evolution [54]. Class II insulators are those bound by Su(Hw) [52], and unlike Class I insulators, Su(Hw) does not colocalize with Pol II [55]. Instead, Su(Hw)-associated insulators recruit chromatin remodeling complex Brahma and histone acetyltransferase complex SAGA, resulting in depletion of nucleosomes in *Drosophila* cell culture, and Su(Hw) also recruits origin recognition complex (ORC) to help initiate gene transcription [55]. 

One of the most well-studied insulator sequences is the *gypsy* retrotransposon. The *gypsy* insulator is named after its localization to the 5′ untranslated region of the *gypsy* retrotransposon. Two proteins fundamental to the *gypsy* insulator are Su(Hw) and Mod(mdg4) [56]. The *gypsy* insulator sequence has 12 places for Su(Hw) to bind, making the *gypsy* complex a class II insulator [57]. Centrosomal protein 190 (CP190) was later discovered to bind both Su(Hw) and Mod(mdg4) and is the third required component of the insulator complex [39]. Su(Hw) contains twelve zinc finger domains and interacts with Mod(mdg4) through its C-terminal region [58]. CP190 and Mod(mdg4) can interact through the BTB domain of Mod(mdg4) and the M domain of CP190 [58]. One of Mod(mdg4)’s domains that is rich in glutamine can interact with Su(Hw)’s N-terminal domain [58]. CP190 also contains a BTB domain, through which it can interact with two adjacent sites on the N-terminus of Su(Hw) [59].

These three insulator proteins interact with many other proteins as well. Mod(mdg4) interacts with Zeste through C-terminal and BTB domains, which appears to be required for enhancer–promoter communication of the *white* gene [60]. Enhancer of *yellow* 2 (E(y)2) also interacts with Su(Hw) and is required for barrier function of the 1A2 *gypsy* insulator [61]. This insulator activity is critical for proper expression of the *yellow* gene [62]. Heterochromatin protein 1 (HP1a) interacts with HIPP1 and associates with various insulator proteins including Su(Hw), CP190, and Mod(mdg4) [63]. However, interactions of HIPP1 and insulator complexes appeared to be distinct from interactions between HP1a and HIPP1 in heterochromatin [63]

Su(Hw), Mod(mdg4), and *gypsy* insulator sequences are found at the periphery of the nucleus, and it has been shown that inserting the *gypsy* insulator sequence causes a section of chromatin to relocate from the center of the nucleus to the periphery [64]. *gypsy* sequences also come together in long-distance interactions at the same location in the nucleus [64]. The *gypsy* insulator forms a loop, with the *gypsy* motifs and Su(Hw) binding sites located at the base of the loop, which was first suggested by Byrd and Corces in 2003 [65]. They also described the nuclear matrix as a skeletal network for the insulators to attach to and that it is necessary for loop formation, and they concluded that the *gypsy* insulator (primarily Su(Hw) and Mod(mdg4)) forms loops by attaching to the nuclear matrix [65].

A second well-studied insulator complex is the late boundary complex (LBC). The LBC is a large (>700 kDa) and more recently discovered complex that binds to *Fab-7* sequences flanking the bithorax complex (BX-C). Architecture proteins CLAMP and GAF seem to depend on each other for binding to the *Fab-7* boundary of the BX-C [66]. The LBC itself includes Mod(mdg4), GAF, and E(y)2 [67]. Furthermore, the LBC only seems to be necessary for the late embryonic development and adult stages of *Drosophila* [67] . The BX-C is one of the HOX gene complexes and is epigenetically silenced via a polycomb-repressed condensed TAD and polycomb response elements (PREs) that are in close proximity to each other, possibly reflecting the organization of the compacted structure by the PREs themselves contacting each other [68,69,70]. ELBA is another insulator complex that regulates the *Fab-7* boundary during early development [71]. It is made up of three proteins: Elba1, Elba2, and Elba3, all of which are required for DNA binding and insulator activity [71]. BEN domains in Elba1 and Elba2 are responsible for DNA binding, and Elba3 is required for holding the two BEN domains together [71]. dCTCF, like the LBC, is also important for the correct regulation of the BX-C [72]. dCTCF regulates Fab-8 and expression of Abd-B in the bithorax complex [34]. 

The LBC has also been shown to bind to male-specific lethal (MSL) recognition sequences on the X chromosomes of males, including *rox1* and *rox2* [66]. *rox1* and *rox2* encode RNAs that are expressed in all somatic cells in male flies, localize with other MSL proteins along the X chromosome, and are likely required for the process of dosage compensation [73]. A study by Ramírez et al. indicated that TAD boundaries on the X chromosome correlate strongly with MSL complex binding sites in males [74]. Furthermore, the 3D organization of the X chromosome is very similar between males and females; therefore, the MSL complex likely does not alter the chromosome architecture, but merely acts upon what is already established [48]. CLAMP also interacts with *rox1* [75] and has been shown to be associated with both the LBC and the *gypsy* complex [42,66]. Chromator appears to be a multifunctional protein that is associated with the MSL complex [76] and regulates polytene chromosome structure by direct association with JIL-1 kinase [77]. 

**Table 1:** Proteins involved in *Drosophila* TAD architecture. Gene names were acquired from FlyBase [78].

Drosophila Gene Name	Alternative Name(s)	DNA Binding Activity	Interactions with Other Architectural Proteins
Boundary element-associated factor of 32kD (BEAF-32)	BEAF-32A/BEAF-32B (isoforms of BEAF-32), BEAF, boundary element-associated factor	Yes via a BED finger domain [79,80]	Chromator [81] CP190 [81]
Centrosomal protein 190kD (Cp190)	Rb188, DMAP190, Cen190, CP-190, Centrosomal Protein 190, CP190	Yes, via zinc finger domains [39]	BEAF-32B [81] Chromator [81] CLAMP [42] dCTCF [82] HIPP1 [63] Mod(mdg4) [39] Pita [51] Su(Hw) [39] ZIPIC [51]
Chromatin-linked adaptor for MSL proteins (Clamp)	CLAMP	Yes, via zinc finger domains [83]	CP190) [42] Mod(mdg4) [42] Su(Hw) [42]
Chromator (Chro)	Chriz, Chro(Chriz)	None found	BEAF-32B [81] CP190 [81] dCTCF [84] JIL-1 kinase [77] Mod(mdg4) [85]
CTCF	dCTCF, CCCTC-binding factor	Yes, via zinc finger domains [34]	Chromator [84] CP190 [82] E(y)2 [86] HIPP1 [63]
deformed wings (dwg)	Zw5, l(1)zw5, zw-5, EG:95B7.6 , zeste-white 5	Yes, via zinc finger domains [50]	(itself) [87]
enhancer of yellow 2 (E(y)2)	ENY2, late boundary complex, LBC	None found	dCTCF [86] GAF [45] Su(Hw) [61]
HP1 and insulator partner protein 1 (HIPP1)	N/A	None found	CP190 [63] dCTCF [63] Pita [63] Mod(mdg4) [63] Su(Hw) [63]
modifier of mdg4 (Mod(mdg4))	E(var)3-93D, doom, mnm, Mod(mdg4)2.2, Mod(mdg4)-67.2	Not directly [43]	Chromator [85] CLAMP [42] CP190 [39] GAF [44] HIPP1 [63] Su(Hw) [56,88]
Motif 1 Binding Protein (M1BP)	N/A	Yes, via zinc finger domains [47]	CP190 [49] Mod(mdg4) [49] Su(Hw) [49]
pita	Spdk, spotted dick	Yes, via zinc finger domains [89]	CP190 [51] HIPP1 [63] ZIPIC [51]
suppressor of Hairy wing (su(Hw))	suHw	Yes, via zinc finger domains [90]	CLAMP [42] CP190 [39] E(y)2 [61] HIPP1 [63] Mod(mdg4) [56,88]
Trithorax-like (Trl)	GAF, GAGA, GAGA factor, Nc70F, GAGA-factor	Yes, via a zinc finger domain [91]	E(y)2 [45] Mod(mdg4) [45]
zelda (zld)	vfl, vielfaltig, EP134	Yes, via zinc finger domains [92]	Ubx [93]
Zinc-finger protein interacting with CP190 (ZIPIC)	N/A	Yes, via zinc finger domains [94]	CP190 [51] Pita [51]

## 3. Interhomolog Interactions

Just as chromosomes can interact with themselves to organize into TADs, they are also subject to complex associations with their homolog. These associations drive how homologous chromosomes pair with one another, complement each other’s transcription regulation in *trans*, and form discrete territories within the nucleus. Interhomolog interactions are genetically and cytologically detectable in *D. melanogaster* because the chromosomes regularly pair with their homolog in somatic tissues. While interactions between homologs are also critical—and perhaps even more so—during meiosis, in this work, we will limit our scope to what is known about homologous chromosome pairing in somatic tissue (see Rubin et al. 2020 for a recent review of interhomolog interactions during meiosis) [95].

Homologous chromosome pairing in *Drosophila* somatic tissue was first visualized by Stevens and then Metz [96,97] in pre-meiotic mitotic metaphases using classic cytological techniques to fix the tissue and stain for the chromosomes. Today, the physical interaction of homologs can be observed at high resolution using methods such as fluorescent in situ hybridization (FISH) or Hi-C. Additionally, the functional consequences of interhomolog interactions, particularly regulatory regions on one homolog being able to influence the expression of a target on the other, can be read out genetically. Below, we review both of these types of interhomolog interactions insofar as they have been investigated in *D. melanogaster*, followed by a discussion of how these interhomolog interactions drive chromosome territory formation within the nucleus.

### 3.1. Homologous Chromosome Pairing in Somatic Tissue

**Figure 2:**
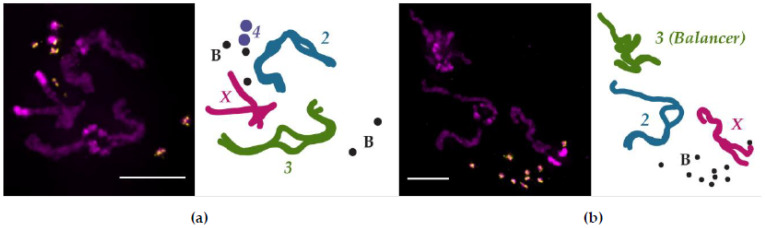
Homologous pairing in *Drosophila melanogaster* somatic cells that carry B chromosomes. (**a**) Tight homologous chromosome pairing between condensed chromosomes is retained primarily in the euchromatic regions. Left, metaphase chromosomes from third-instar larval neuroblasts; right, cartoon trace of each chromosome. The B chromosomes do not appear to pair. (**b**) Homologous chromosome pairing of a balancer chromosome (Chromosome 3), cartooned in green. The other large chromosomes exhibit normal pairing. Chromosome 4 not shown. Magenta: DNA, stained with DAPI. Yellow: fluorescent FISH probe recognizing the *AAGAT* satellite repeat present on Chromosome 4 and the B chromosomes. Scale bar = 5 μm [100].

Cytogenetic methods to examine somatic chromosome pairing in *Drosophila*, similar to those employed by Stevens and Metz, can occasionally capture cells with condensed chromosomes that have yet to fully unpair homologs [96,97,98,99]. An example of the results from this technique is shown in Figure 2, where it was used to investigate the pairing dynamics of small, supernumerary B chromosomes [100]. These B chromosomes are maintained at a high copy number and are mostly—if not entirely—composed of heterochromatin [101,102], making it difficult to ascertain their pairing aptitude with other methods. As shown in Figure 2a, tight homologous chromosome pairing between condensed chromosomes is retained primarily in the euchromatic regions (the arms) of the X, second, and third chromosomes. The separation of homologs is most apparent in the heterochromatin-rich regions of the larger chromosomes, particularly in the pericentromeric regions, which is consistent with previous cytogenetic pairing observations [98,99]. Both the B chromosomes and Chromosome 4 appear not to be engaged in tight homologous pairing; if heterochromatin dissolves its pairing interactions (or promotes anti-pairing) before euchromatin, then the abundance of heterochromatin on the B chromosomes and Chromosome 4 may explain why the homologs of these chromosomes have separated. Certainly, as this technique is repeated, a clearer picture of B chromosome pairing in somatic tissue will emerge.

Though assessing homolog pairing via cytogenetics can be informative, it is not a feasible approach for broad-spectrum studies of homologous chromosome pairing. The cytogenetic method requires cells to be in mitosis and have clearly condensed chromosomes so that each can be easily distinguished; homolog interactions that occur during interphase or in cells that have poorly condensed mitotic chromosomes would not be detected with this technique. A more modern approach to pairing assessment is the use of fluorescent in situ hybridization (FISH), which uses fluorescently labeled nucleic acid probes that are designed to hybridize with a specific sequence within the genome. When applied to an interphase cell, the FISH probe will bind to its target sequence on both homologs. If the homologs are unpaired at that location, then two FISH foci will be visible; however, the FISH signal from two paired homologs is unresolvable and appears as one focus. Using this technique, it was determined that the somatic pairing of homologs in *D. melanogaster* begins early in development during the embryonic mid-blastula transition, reaching an appreciable frequency of pairing by cycle 14 [103,104,105]. The mid-blastula transition occurs between cycles 10 and 14 and coincides with an elongation of the cell cycle and zygotic gene activation (ZGA) as part of the maternal–zygotic transition [106]. The onset of homologous chromosome pairing, however, appears not to be driven by a specific gene product expressed from the zygotic genome because embryos that were deficient for both copies of entire chromosome arms were still able to initiate pairing [107]. Thus, homolog pairing is likely a facet of the developmental program at large, which is consistent with recent studies examining the formation of TADs and the 3D genome organization that emerges during ZGA [108,109].

Though no one zygotic gene product may be responsible for initiating homolog pairing, we do know of a handful of genes that appear to promote the frequency of homologous chromosome pairing when disrupted in other tissues and in cell culture. Not surprisingly, one of these genes is *su(Hw)*, which also plays a critical role in TAD formation (as discussed above). In the absence of Su(Hw), homologous chromosome pairing in the imaginal discs is reduced by around 30% [110]. Another gene that has been shown to be important for homolog pairing is *Topoisomerase 2* (*Top2*), which is a type II topoisomerase that can make double-strand breaks and catenate and decatenate DNA. Disruption of Top2, either via RNAi knockdown or treatment with the topoisomerase inhibitor ICRF-193, results in a significant reduction in homolog pairing [99]. Despite its ability to directly alter the DNA topology, the pairing defects observed when the Top2 function is impaired may instead be related to its role in stabilizing Su(Hw) insulators by preventing the degradation of an isoform of Mod(mdg4) [111]. 

Just as there are genes that play a role in promoting homologous chromosome pairing, some genes appear to have the ability to antagonize it. Condensins are multi-unit, ring-like protein complexes that laterally (condensin I) and axially (condensin II) organize chromosomes [112]. A component of condensin II, Cap-H2, has been shown to be necessary for the disassembly of nurse cell polytene chromosome pairing during oogenesis, as well as sufficient to disrupt the pairing between the polytene chromosomes in salivary gland cells [113]. Activity of condensin II is negatively regulated by SCF^Slimb^, a ubiquitin ligase complex that targets Cap-H2 for degradation [114]. The anti-pairing action of condensin II is likely due to its promotion of axial compaction of chromatin as facilitated by the interaction between Cap-H2 and Mrg15, the latter of which acts as a loading factor [115,116].

In an effort to identify additional genes that may play a role in homologous chromosome pairing, two separate studies conducted FISH-based RNAi screens in *Drosophila* cell culture. Both screens were a great success (59 hits for pairing-promoting genes [117], 40 and 65 hits for pairing-promoting and anti-pairing genes, respectively [118]), and the broader conclusion that arose from these studies was that most of the hits have functional roles that are tied to cell cycle events. This connection of homolog pairing and the cell cycle is consistent with previous reports of pairing status changes through cell cycle progression [5,99,104]. 

An even more sensitive method than FISH has recently emerged to examine homolog pairing on a genome-wide scale. Conventional Hi-C cannot determine whether DNA fragments were derived from the maternal or the paternal homolog; therefore, it is not possible to differentiate interhomolog interactions from inter-sister interactions. Utilizing single-nucleotide variants (SNVs) to establish the parental haplotype, a pair of studies were able distinguish maternal from paternal chromosomes, leading to a contact map that included interhomolog interactions in early embryos [119] and clonal cell lines [120]. These studies found that interhomolog interactions varied in strength across the genome, and that pairing correlated strongly with active chromatin.

These haplotype-resolved Hi-C studies also showed that pairing was not uniform across the genome and that TADs are organized intrachromosomally but participate in homolog pairing via TAD boundaries, two observations that fit with the button model of how homologs pair (Figure 1) [121]. Indeed, this model explains why a multiply inverted balancer chromosome both has mostly (88%) unchanged TAD boundaries [122] and can still pair with its homolog, albeit with more contortion (Figure 2B). The button model further predicts that pairing initiates at insulator-enriched TAD boundaries, but what is driving this organization? At least in early development, the sites that were found to be the most frequently paired were also enriched with Zelda (Zld). Zld, a zinc finger protein that mediates chromatin accessibility, is a key activator of transcription during ZGA [92] and is enriched at domain boundaries that are transcriptionally active [108]. This result is consistent with the observation that Zld helps establish TAD boundary insulation [108], and that insulators can act to stabilize interhomolog interactions [123,124]. Together, these studies indicate that Zld-mediated transcription during development is important for the establishment of early TAD domain boundaries, which serve as sites of robust homologous chromosome paring.

### 3.2. Transvection and Interhomolog Communication

Somatic homologous chromosome pairing results in some interesting functional consequences. Given a situation where one homolog carries a promoter and its corresponding coding region but lacks the necessary enhancer element, and on the other homolog, the enhancer element is present but the promoter and coding region it regulates is absent, classical genetics would predict that no gene product could be produced since neither homolog carries all three necessary components. In *Drosophila*, this is not necessarily the case due to the phenomenon of transvection [125]. The pairing of homologs in somatic tissues enables regulatory elements on one homolog to exert their effects on the opposite homolog, resulting in genetic complementation in *trans* and the normal expression of a gene. Several genes have been observed to be subject to *trans* effects in *Drosophila*, including *Ultrabithorax (Ubx)* [125], *yellow* (*y*) [126,127], *decapentaplegic* (*dpp*) [128], *cubitus interruptus (ci)* [129], *brown (bw)* [130], *engrailed (en)* [131], *eyes absent (eya)* [132], *Gpdh* [133], *hedgehog (hh)* [134], *pointed (pnt)* [135], *sex combs reduced (scr)* [136], TMR of Abd-B [137], *spineless (ss)* [138], *vestigial (vg)* [139], *white (w)* [140], and *wingless (wg)* [141].

Transvection is a type of interhomolog communication that appears to be a fortunate consequence of position-dependent homologous chromosome pairing, while other examples of interhomolog communication have been found that are not position-dependent [138] and that can occur over a long distance and be cell-type specific [142,143]. Though the local chromatin architecture may influence the level of transvection output [144], the requirement of pairing and homology in transvection is well established [126,145,146,147,148], and it appears that any enhancer can likely act on any promoter provided the two are at the same genomic position [149]. Since pairing is likely mediated by insulators, it is not surprising that insulators also play a key role in transvection. As demonstrated at the *yellow* locus, two paired *gypsy* insulators on each homologous chromosome facilitated the *trans* activation of the *yellow* promoter, implicating the *gypsy* insulator binding protein Su(Hw) in facilitating homologous pairing [150]. This study was echoed by a similar investigation that demonstrated *gypsy* insulators that flanked a reporter construct supported transvection [123]. Recently, additional studies have made it undeniably clear that insulators are required for transvection [121,151]. 

## 4. Chromosome Territories and the Rabl Configuration

With the complexity of organization at the chromosome level, it is to be expected that the genome within the nucleus is also highly organized. In *Drosophila* and in many other organisms, each chromosome occupies a discrete territory [152]. This arrangement was first suggested by Rabl [153] and later expanded on by Boveri, who introduced the term chromosome territory (CT) [154]. Modern methods have clearly demonstrated the presence of CTs in *Drosophila* using both cytological [155] and genomic [156] approaches. One benefit of CTs may be to protect the genome from aberrant repair products after suffering damage, as a loss of territory formation can lead to an increase in chromosome rearrangements [157]. 

Within each of these territories, pericentromeric heterochromatin has been shown to be hierarchically organized and forms into its own territories that remain near to its corresponding chromosome territory [158]. This result is consistent with chromosomes being held in a Rabl configuration, which is when the centromeres are held together at one end of the nucleus and the telomeres are positioned at the opposite end (Figure 1). Analysis by FISH confirms that chromosomes adhere to the Rabl configuration [159], as do advanced genomic methods that integrate Hi-C data with lamina-DamID experiments [156] or visualize chromosome contacts for maternal and paternal homologs in haplotype-resolved Hi-C [119]. 

## 5. Conclusions

From TADs to territory, the genome of *Drosophila melanogaster* is highly organized. Interphase chromatin is looped and folded to bring distant modulators into proximity with the genes they affect. This intrachromosomal organization is reliably re-established cell cycle after cell cycle. In *Drosophila*, an additional layer of organization operates through interhomolog interactions, where regions of one homolog can pair with the corresponding region on the other homolog. This pairing is mediated by proteins that also play a role in TAD formation, suggesting that the multi-scale organization we observe may be the result of a unified protein network of genome architects.

Equipped with the ability to assay chromosome contacts both genome-wide and in combination with other layers of genome data, the next decade of chromosome dynamics is sure to please. It will be exciting to investigate how genome organization changes in relation to various other critical events, such as metamorphosis and gametogenesis. One interesting question is how pairing in somatic tissue compares to the pairing observed in the female germline, and if polar mutants that affect the distribution of crossovers also affect genome organization. Another question is how small, supernumerary chromosomes pair and organize within the nucleus. The B chromosomes are less than 2 Mbp in size and do not carry euchromatic regions [101]; therefore, the ability to pair via active chromatin within TAD boundaries is not an option. Perhaps homologous chromosome pairing has persisted due to its ability to modulate gene expression in *trans*; therefore, the lack of pairing of B chromosomes may not be consequential. Additionally, it will be interesting to uncover what mediates the homology search in interhomolog interactions. What ensures an insulator protein at one locus on a homolog is interacting with the insulator protein that is bound to the corresponding locus on the other homolog? We know that pairing proceeds by a random walk model [104], and that non-coding RNAs help regulate genome organization [160]; therefore, perhaps a chromosome’s motion and non-coding RNAs combined with the accessibility of chromatin at pairing sites will be the connection. Regardless, this is an exciting time for investigations into the organization of the genome.
